# Effect of Hereditary Hemochromatosis Gene H63D and C282Y Mutations on Iron Overload in Sickle Cell Disease Patients

**DOI:** 10.4274/tjh.2015.0254

**Published:** 2016-12-01

**Authors:** Yunus Kasım Terzi, Tuğçe Bulakbaşı Balcı, Can Boğa, Zafer Koç, Zerrin Yılmaz Çelik, Hakan Özdoğu, Sema Karakuş, Feride İffet Şahin

**Affiliations:** 1 Başkent University Faculty of Medicine, Department of Medical Genetics, Ankara, Turkey; 2 Başkent University Faculty of Medicine, Department of Hematology, Ankara, Turkey; 3 Başkent University Faculty of Medicine, Department of Radiology, Ankara, Turkey

**Keywords:** Hemochromatosis, HFE gene, iron overload, p.C282Y, p.H63D, sickle cell anemia

## Abstract

**Objective:**

Hemochromatosis is an autosomal recessive disease that is one of the most important reasons for iron overload. Sickle cell disease is a hemoglobinopathy that occurs as a result of a homozygous mutation in the hemoglobin gene. Erythrocyte transfusion is frequently used in the treatment of this disease. Iron overload as a result of transfusion is important in the mortality and morbidity of sickle cell anemia patients as well as in other hemoglobinopathies. In this study, the effect of hemochromatosis gene (HFE) p.H63D and p.C282Y mutations on transfusion-related cardiac and liver iron overload in sickle cell disease patients who carry homozygous hemoglobin S mutation has been investigated.

**Materials and Methods:**

This is a prospective single-center cross-sectional study in patients with homozygous hemoglobin S mutation between the years 2008 and 2013. The patients were divided into two groups. The first group (group A, n=31) was receiving chelation therapy and the second group (group B, n=13) was not. Direct and indirect iron loads were analyzed by magnetic resonance imaging and biochemically, respectively. HFE gene mutations were analyzed by polymerase chain reaction-restriction fragment length polymorphism method. Statistical analyses were performed by independent samples t-test.

**Results:**

p.H63D mutation was detected in 10 (32.3%) patients in group A and in only 1 patient (7.7%) in group B. When the 2 groups were compared for iron overload, iron deposition in the liver was significantly higher in group B (p=0.046). In addition, in group A, iron deposition was significantly higher in HFE mutation carriers compared to patients without the mutation (p=0.05).

**Conclusion:**

Results of this study showed that HFE gene mutations are important in iron deposition in the liver in patients with sickle cell disease.

## Abstract (tr)

**Amaç:**

Hemokromatozis, demir birikiminin önemli nedenlerinden biri olan otozomal resesif bir hastalıktır. Orak hücreli anemi, hemoglobin genindeki homozigot mutasyon sonucu ortaya çıkan bir hemoglobinopatidir. Eritrosit transfüzyonu, bu hastalığın tedavisinde sıklıkla kullanılmaktadır. Transfüzyonun yarattığı demir yükü diğer hemoglobinopatilerde olduğu gibi orak hücreli anemi hastalarının mortalite ve morbiditesinde önem kazanmaktadır. Bu çalışmada hemokromatozis geni (HFE) p.H63D ve p.C282Y mutasyonlarının, homozigot hemoglobin S mutasyonu taşıyan orak hücreli anemi hastalarında, kalp ve karaciğerde transfüzyonla ilişkili demir yüklenmesine olan etkisi araştırılmıştır.

**Gereç ve Yöntemler:**

Bu çalışma, homozigot hemoglobin S mutasyonu olan hastalarda 2008-2013 yıllarını kapsayan prospektif, tek merkezli kesitsel bir çalışmadır. Hastalar şelasyon tedavisi alan (n=31) ve almayan (n=13) olarak iki gruba ayrıldı. Hastalarda direk ve endirekt demir yükü sırasıyla manyetik rezonans görüntüleme ve biyokimyasal olarak analiz edildi. HFE geni mutasyon analizi polimeraz zincir reaksiyonu-restriksiyon fragment uzunluk polimorfizmi yöntemleri ile gerçekleştirildi. İstatistik analizi Independent samples t-testi uygulanarak gerçekleştirildi.

**Bulgular:**

p.H63D mutasyonu grup A’da 10 hastada (%32,3), grup B’de ise sadece 1 (%7,7) hastada saptandı. Demir birikimi açısından gruplar karşılaştırıldığında karaciğerde demir birikiminin grup B’de istatistiksel olarak anlamlı derecede yüksek olduğu görülmüştür (p<0,05). Grup A’da, mutasyonu olan bireylerde olmayanlara göre karaciğerdeki demir birikiminin istatistiksel olarak anlamlı derecede yüksek olduğu görülmüştür (p=0,05).

**Sonuç:**

Bu çalışmanın sonucu HFE genindeki mutasyonların, orak hücreli anemi hastalarında karaciğerde demir birikimi üzerinde etkili olduğunu göstermektedir.

## INTRODUCTION

Hereditary hemochromatosis (HH) is an autosomal recessive disease that is one of the important reasons for transfusion-unrelated iron deposition [1]. The hemochromatosis (HFE) gene, encoding a transferrin receptor binding protein that regulates iron absorption from the intestine, is responsible for the disease and its point mutations result in increased iron absorption and accumulation [2,3].

The penetrance of the disease is low, as only 1% of p.C282Y homozygous individuals have clinical presentations. The disease phenotype results from primary or secondary causes. Primary (hereditary) hemochromatosis is usually due to gene mutations including the HFE gene as well as other genes including transferrin receptor-2 and ferroportin. Secondary hemochromatosis is a result of inherited or acquired anemia requiring frequent erythrocyte transfusions [1]. The hereditary causes of secondary hemochromatosis include thalassemia, hereditary spherocytosis, and sideroblastic anemia, and the acquired diseases include anemia due to blood loss [1].

Sickle cell anemia is a hemoglobinopathy resulting from a homozygous point mutation in the hemoglobin gene characterized by sickling of erythrocytes [4]. Sickling results in vaso-occlusion, hemolysis, and chronic anemia, which results in increased cardiac output due to volume overload and hypoxia as a result of vaso-occlusion, which ends with organ dysfunction [5]. Erythrocyte and blood transfusions are frequently used in the treatment of the disease. Transfusion-related iron overload is important in mortality and morbidity of sickle cell anemia patients like in other hemoglobinopathies [3,6]. Mutation frequencies are known to be different between ethnic groups.

In the current study, the relationship between HFE gene p.H63D and p.C282Y mutations and iron deposition occurring during sickle cell anemia progress and their effect on cardiac and liver iron overload have been investigated.

## MATERIALS AND METHODS

### Patients

The study was performed as a prospective, single-center, cross-sectional study on homozygous hemoglobin S mutation patients followed in the adult hematology department between 2008 and 2013. A total of 45 patients aged between 20 and 42 years were enrolled in the study and divided into two groups according to administration of chelation treatment. Patients in group A (n=31) were receiving chelation treatment and those in group B (n=13) were not. There were 21 male and 10 female patients in group A and 4 male and 9 female patients in group B. Patients in group A received deferasirox (Exjade, Novartis, Switzerland) therapy when they had evidence of chronic transfusional iron overload. This evidence included the transfusion of at least 100 mL/kg of packed red blood cells, or a serum ferritin level consistently greater than 1000 µg/L. Initial daily dose was 20 mg/kg, per os. All patients required escalation of 5 to 10 mg/kg per daily dose to keep serum ferritin from consistently falling from baseline. If the serum ferritin fell below 500 µg/L, the therapy was interrupted. Duration of therapy was 30 months (range: 18-44 months).

Patients with contraindications for magnetic resonance imaging (MRI) were excluded from the study. Clinical and laboratory information of the patients was obtained from the hospital information management system (Nucleus v9.3.39, Monad Ltd., Ankara, Turkey).

### Hematological and Biochemical Analyses

Blood cell count and aspartate aminotransferase and alanine aminotransferase levels were analyzed by automatized methods in the laboratory. Serum iron concentration (normal range: 59-158 µg/dL), transferrin saturation (normal range: 15%-75%), serum ferritin (normal range: 40-340 ng/mL for males and 14-150 ng/mL for females), and C-reactive protein levels were detected by enzyme-linked immunosorbent assays.

### Magnetic Resonance Imaging Analyses

All imaging analyses were performed as described previously with slight modifications, and a 1.5T MRI system was used for these analyses (Avanto, Siemens, Erlangen, Germany) [7,8,9]. Briefly, liver and myocardial measurements included T2* value screenings. Screening time was 14 s. The scan duration was 14 s. The T2* of the heart was assessed by a cardiac gated single breath-hold multiecho technique. Midventricular short-axis images were obtained using a gradient-echo sequence (FOV, 440 mm; TR, 120 ms; TE, 3.0-21.7 ms [8 echo times]; flip angle, 20; slice thickness, 10 mm; matrix, 256x104; number of averages, 1; bandwidth in Hz/pixel, 814). To measure the liver iron concentration (LIC), phased-array torso coils were used for signal detection. The lung was excluded on the axial plane as much as possible. Liver T2* values were assessed by single breath-hold multiecho technique. Axial images through the liver were obtained using a gradient-echo sequence (FOV, 400; TR, 120 ms; TE, 4.3-20.2 ms [6 echo times]; flip angle, 20; slice thickness, 10 mm; matrix, 256x80; number of averages, 1; bandwidth in Hz/pixel, 814). T2* measurements were performed with Thalassemia Tools (Cardiovascular Imaging Solutions, London, UK). A full-thickness region of interest was drawn in the interventricular septum. The signal intensity of this region for each echo time was measured and plotted as an exponential signal decay curve. The lower limit of normal for T2* in the detection of myocardial iron deposition has been reported as 20 ms, and this value was used as the cut-off in this study [7,8,9]. A T2* value of >20 ms indicated no cardiac iron overload, and ≤20 ms indicated cardiac iron overload [10]. Liver iron deposition was evaluated by R2* value (R2*=1000/T2*). The R2* value was converted to a liver biopsy equation by using the calibration curve drawn during the study [11]. LIC in dry tissue of >1.6 mg Fe/g was regarded as hepatic siderosis.

### HFE Gene p.H63D and p.C282Y Mutation Analyses

**Figure 1:**
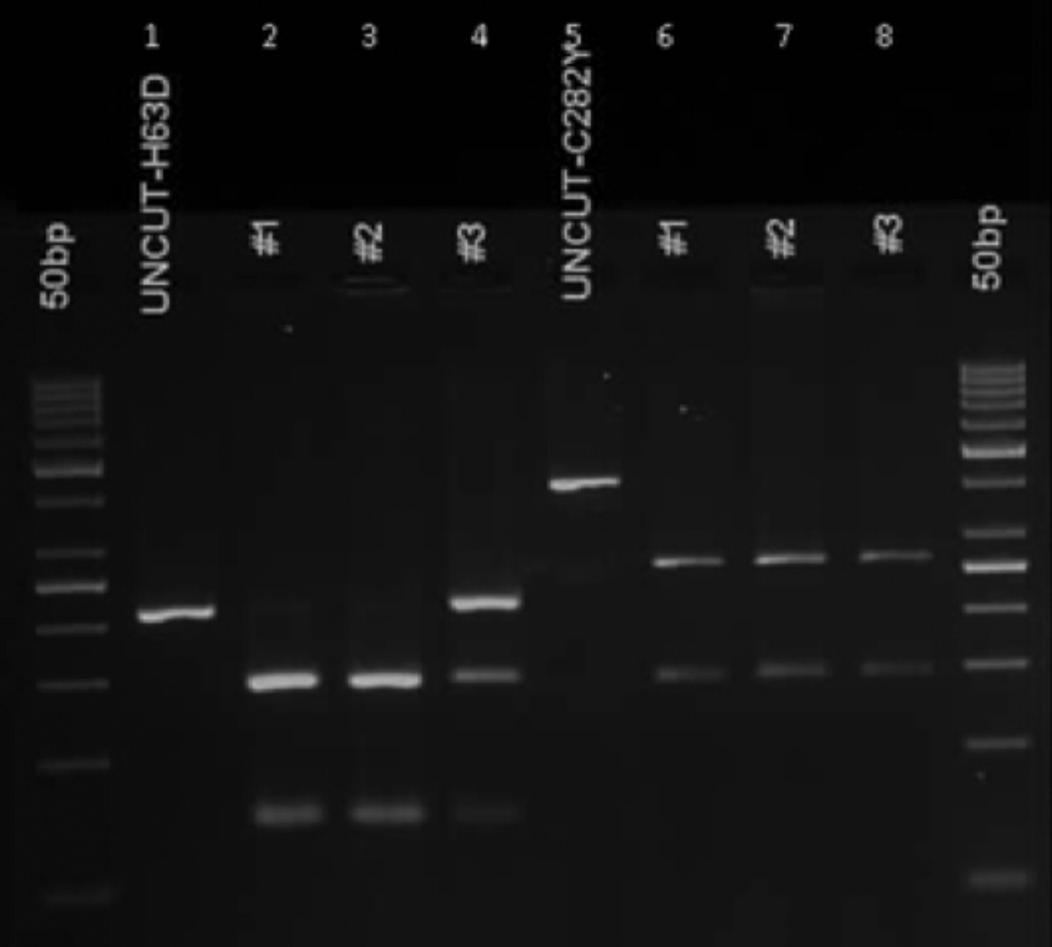
Gel image of H63D and C282Y mutations analyzed by polymerase chain reaction-restriction fragment length polymorphism. Lanes 1 and 5 are uncut polymerase chain reaction products, lanes 2 and 3 are samples from patients normal for H63D mutation, and lane 4 is a heterozygous patient sample. Lanes 6-8 are normal patient samples for C282Y mutation.

DNA isolation was done from peripheral blood samples of the patients who were included in the study and signed the informed consent form. HFE gene p.H63D and p.C282Y mutations were analyzed by polymerase chain reaction (PCR)-restriction fragment length polymorphism. Primer sequences and product sizes for p.H63D and p.C282Y mutations are shown in Table 1. PCR conditions were 15 min at 95 °C for initial denaturation, followed by 35 cycles of 45 s at 94 °C, 30 s at 58 °C, and 30 s at 72 °C. The PCR was completed after a final elongation step of 7 min at 72 °C. PCR products were digested with BclI and RsaI restriction endonucleases for H63D and C282Y mutation analyses, respectively. The band lengths after digestion are shown in Table 1. A gel image of the digested products is shown in Figure 1.

### Statistical Analysis

The Kolmogorov-Smirnovtest was used to show the normal distribution of the data. Significant differences between groups were determined using t tests. Data were expressed as means. All statistical analyses and tests were performed with the SPSS statistical package (SPSS 17.0, Chicago, IL, USA) and p<0.05 was regarded as statistically significant.

## RESULTS

A total of 45 patients aged between 20 and 42 years were enrolled in the study. There were 20 male and 11 female patients in group A and 5 male and 9 female patients in group B. All patients were homozygous for the hemoglobin S mutation. Biochemical and MRI results of group A and group B patients are shown in Table 2. When the 2 groups were compared for iron deposition in the liver, iron deposition was found to be significantly lower in group A (p=0.05). In addition, platelet count was found to be significantly higher in group A (p<0.03). We did not observe a statistically significant difference between the 2 groups when other MRI and biochemical values were compared.

HFE gene H63D mutation was detected in 10 (32.3%) patients in group A and in 1 (7.7%) patient in group B (Table 3). In group A, liver iron deposition was significantly higher in patients with mutations compared to the patients without mutations (p=0.05) (Table 4). C282Y mutation was not observed in any of the patients included in the study (Table 3).

## DISCUSSION

Humans do not have a physiologic mechanism to excrete excess iron absorbed from the intestine. Iron metabolism is strictly controlled by intestinal absorption [12]. In the case of increased iron absorption, iron deposits occur in all organs. As iron accumulation is a problem directly influencing the prognosis in sickle cell disease patients, we proposed that coexisting HFE mutations could contribute to the deposition process in these cases.

HH is characterized by hepatic fibrosis, cirrhosis, diabetes, skin pigmentation, hypogonadism, and articular and cardiac disorders and, in advanced stages of the disease, iron deposition in other organs as a result of increased iron absorption from the intestines [10]. The disease occurs as a result of HFE gene H63D and C282Y mutations [13].

Sickle cell anemia is one of the most frequent hereditary anemias resulting from a homozygous point mutation in the hemoglobin gene [14]. Endothelial cell activation and microvascular ischemia may cause tissue damage in sickle cell anemia, and the spectrum of clinical outcomes and tissue damage severity varies among individuals. Because of the above findings, it was suggested that although sickle cell anemia is a single-gene disease, it should be assessed as a multifactorial disorder [15]. Blood transfusion and blood change, used frequently in the treatment of the disease, cause a decrease in erythrocyte number and sickle cell hemoglobin polymer formation. However, as a result of treatment, iron deposition and organ damage occur in patients [12,16]. In our study, the relationship between iron deposition and HFE gene H63D and C282Y mutations has been investigated. A total of 45 patients were enrolled in the study.

HFE gene mutations have been investigated in another hemoglobinopathy, thalassemia, and the presence of a single mutation was not found to affect iron overload [3]. The effect of the presence of these mutations has been also investigated in sickle cell anemia and they were not found to affect the degree of iron overload [13,14].

C282Y mutation was observed in 90% of hemochromatosis patients previously [10]. We did not observe this mutation in our patients. On the other hand, we observed H63D mutation in a heterozygous state in 9 patients (29%) and in a homozygous state in 1 (3%) patient in group A. The mutation was observed in a heterozygous state in only 1 patient in group B (Table 3). The effect of H63D mutation on iron deposition has not yet been clearly identified. Iron deposition in the liver was found to be significantly higher in group B (p<0.05). In the literature, in some sickle cell patients who received chelating agents, iron deposition in tissues was observed, whereas in others it was not [1,4]. Our results show that genetic backgrounds of patients affect the results of the treatment and clinical benefits from treatment.

The C282Y mutation has been reported to be more effective in iron absorption equilibrium than the H63D mutation [2,10]. As we did not find the C282Y mutation in our patients, we concluded that the H63D mutation could also be effective on iron absorption even in the heterozygous state.

Although determination of ferritin level is an indirect method, it is one of the most valuable tools for follow-up of iron overload in patients with hemoglobinopathy. The source of the ferritin in the blood may be different. In the case of high levels of ferritin (3000 µg/L), the possible source is blood and bone marrow; however, if the measurable level of ferritin is below 3000 µg/L, the possible source of ferritin is the reticuloendothelial system. Fluctuation of the measured ferritin level may be observed in the case of infection or inflammations. It has already been shown that the most accurate indicator of total body ferritin load is liver ferritin level [9]. Although liver biopsy was not performed for the patients to determine the ferritin load of the liver, and this may be considered as a weakness of the study, MRI is one of the other valuable tools to determine ferritin load in the liver and heart, and reproducibility is one of the strong features of this method [11].

## CONCLUSION

HFE gene mutations are effective on iron deposition in the liver in sickle cell disease patients. In patients for whom recurrent erythrocyte transfusions are required, genotyping of the HFE gene will be helpful while management with chelating agents is being planned.

## Acknowledgments

This study was approved by the Başkent University Institutional Review Board (Project No: KA09/254) and supported by the Başkent University Research Fund.

## Ethics

Ethics Committee Approval: This study was approved by Başkent University Institutional Review Board (Project no: KA09/254);

Informed Consent: Written informed consent was obtained from all patients.

## Figures and Tables

**Table 1:**
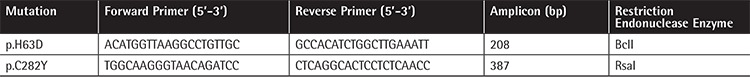
Primer sequences, amplicon lengths, and restriction enzymes used in the study.

**Table 2:**
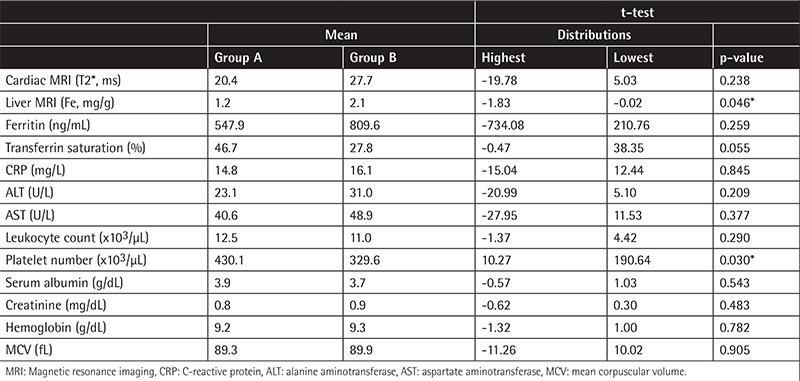
Biochemical and magnetic resonance imaging results of patients in group A and group B. Liver iron deposition and platelet count were found to be significantly different in group A compared to group B (*p<0.05).

**Table 3:**
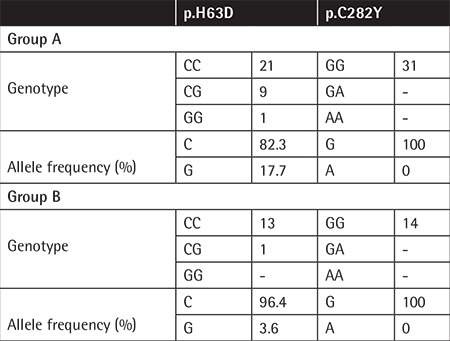
Genotype and allele frequencies of patients in the 2 groups.

**Table 4:**
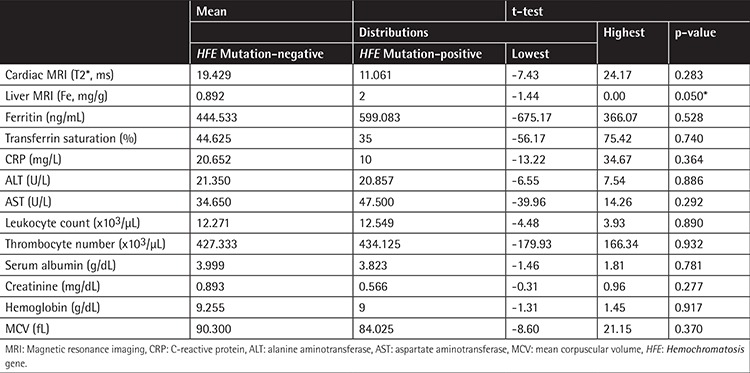
Biochemical and magnetic resonance imaging measurement results of patients in group A. Liver iron deposition was found to be significantly higher (*p=0.05) in patients with HFE mutations compared to the patients without HFE mutations.
